# 

**Published:** 1910-01-15

**Authors:** 


### SUBJECT INDEX TO VOLUME LXIV.[1]

### EVENT AND COMMENT.

Country Dentists Problem, 1.
Dental Anesthetics, 163.
Dental Graduates for 1909 and 1910 Compared, 433.
National Meetings at Denver, 379
Oral Hygiene Campaign, 166.
Oral Prophylaxis, 542.
Plan for a New National Association, 436.
Professional Ideal, 541.
Professional Education, 271.
Prosthodontia, 109.
Public Health, 487.
Public Ministrations to Poor Children, 55.
Stomatologists vs. Dentists, 325
Close of this Volumn and Promise of Another, 595.
The New National Dental Association, 434.
Will the Gold Filling Come Back, 217.

### FORMAL PAPERS.

Advice in Professional Economic J. P. Root, 517.
An Unusual Condition, L. P. Haskell, 371.
A Period of Stress in Childhood, Vida A. Latham, 543.
Antagonizing Complete Artificial Dentures, Geo. H. Wilson, 132.
A Step Toward a Rational Diet, C. L. Palmer, 439.
Bad Teeth versus Good Health, J. J. McCarthy, 560.
Business Building. W. H. Trueman, 5.
Care of Children's Teeth, T. E. Weeks 512.
Care of New York Childrens' Teeth, H. L. Wheeler, 254.
Clinical Experience with Aschers Enamel, E. F. Lewis, 219.
Comparative Values of Different Silicate Cements, W. B. Cavanagh, 327.
Dental Economics, C. E. Abbott 576.
Dental Dispensaries as Sanitary Educators of the Public, C. R. Lyndstrom,
Dental Hospital, Alice M. Stevens, 140.
Dental Hygiene Council of Massachusetts, W. H. Potter, 57.
Dental Operations that are a Menace to the Teeth, W. H. Whitslar, 394.
Dentists as Buyers, G. W. Clapp, 29.
Derivation of Local Anesthetic Drugs Used in Dentistry, W. H. Y. Collins, 521.
Diseases of the Upper Respiratory Passages and their Relations to Oral Deformities, W. H. Treney, 416.
Educating a Dentist to his Duty, 34.
Electric Mallet, B. J. Maerklein, 234.
Ether an Antidote to Cocaine, J. E. Engstead, M.D., 307.
Extending the Field of Dentistry, "Iconoclast",
Fletcherism and some of its Effects on Nutrition, J. B. West 445.
How shall the Stomatologist be Educated? E. S. Talbott, 329.
How shall the Stomatologist be Educated? J. E. Powers, 341
How shall the Stomatologist be Educated? M. W. Cryer, 347.
Important Dimensions in Artificial Teeth, Geo. W. Clapp, 127.
Improvement of Inhaling Apparatus, J. E. Lumbard, 567.

Important Aphorisms Applied to Plate Work, L. P. Haskell, 471.
Influence of Climate on Dental Caries, A. S. Underwood, 555.
Injection Anesthesia in Conservative Operations, A. H. Parrott, 191.
Injections for Local Anesthesia, M. Polet, 204.
Iodine in Mouth Diseases, E. S. Talbott, 391.
Metabolism and Mouth Disease, H. R. Harrower, 569.
Mistakes, C. T. Warner, 408.
Nitrous Oxide and Oxygen, Rebreathing and Prevention of Shock, W. D. Gatch, 167.
Non-Cohesive Gold Foil Filling, J. V. Conzett, 249.
Notes on Local Anesthetics, John Harper, 187.
Obligations of Universities to Medical Education, H. S. Pritchett, 287.
Painless Operations by Alveolar Injections, B. H. Masseling, 465.
Pathology and Treatment of Alveolar Abscess. Personal Experiences, L. P. Haskell, 136.
Practical Treatment of Mouth Pyorrhea, D. D. Smith, 597.
Practical Treatment of True Pyorrhea Alveolaris, J. P. Buckley, 490.
Present Status of Nitrous Oxide, F. W. Bancroft, 384.
Preventive Dentistry, Dr. Alice G. H. Duden, 502.
Professional Code of Ethics and some of his Privileges, Geo. W. Clapp, 40.
Prolongation of Anesthesia with Hypervolatile Drugs, C. G. Parsons, 304.
Public School Education in Oral Hygiene, P. G. White, 80.
Pyocyanase, Carl Ruttloff, 455.
Pyorrhea Alveolaris as a Cause of Colitis, 314.
Quinine and Urea as a Local Anesthetic, 309.
Relation of Universities to Medical School, J. G. Schurman, 274.
Relief of Pain During Dental Operations, Geo. T. Gregg, 179.
Science of Practice Building, Jas. W. Pogue, 17.
School Childrens Teeth in New South Wales, Wm. Rushton, 68.
Silicate Cements, G. C. Poundstone, 459.
Some Uses for "Dentalone," C. S. Service, 625.
Sulphuric Acid in Pulpectomy, R. Kern, 312.
Testing Gold Fillings, 237.
The Anatomic Occlusion of Artificial Teeth, J. H. Prothero, 112.
The Hand Mallet, Reason for Using it, E. K. Wedelstaedt, 225.
Treatment of Aphthea., 313.
Utilizing Atmospheric Pressure for Retention of Lower Dentures, D. H. Young, 138.
What Constitutes a Cure of Alveolar Pyorrhea, R. G. Hutchinson, Jr., 613.
Young Men Wanted, 413.

OBITUARY.

Frank S. Buckley, 474.
J. Y. Crawford, 316.
Isaac M. Douglas, 141.
Mary Sibine Taft, 142.
Wm. H. Todd, 475.

BIBLIOGRAPHY.

Broomell's, Anatomy and Histology of Mouth, 146.
Compend of Histology, 146.
Brophy's Oral Surgery, 485.
Buckley's Dental Materia Medica Pharmacology and Therapeutics, 142.

Dorland's Medical Dictionaries, 145.
Gray's Anatomy, 486.
Guereni's Dental History, 318.
Koch's History of Dental Surgery, 50.
Illinois Dental Directory, 142.
Prinz's Dental Materia Medica, 97.

### INDENTS.

A Dollar Earned vs. A Dollar Saved, 100.
A Duty of the Dental Profession, 533.
Abscess Remedy, 583.
Aged and Indigent Dentist's Fund, 322.
Alcohol and Dental Caries, 535.
Antrum Treatment, 480.
Aphthous Ulcers, 211.
Arseneus Acid in Painless Obligation, 319.
Attainment of Professional Education, 592.
Bill to Prevent Corporations Doing Professional Work, 321.
Calmine, 633.
Clay Pipes and Gold Inlays, 527.
Cleaning Pyorrhea Pockets, 530.
Dangers in Chronic Alveolar Abscess, 581.
Death Following Tooth Extraction, under Gas, 428.
Dental Prophylaxis Aid, 531.
Dental vs. Medical Requirements, 323.
Dentists Needed, 531.
Dental Caries, 261.
Dentists and Charity, 106.
Dental Plate Work its Professional Place, 150.
Dentistry Legalized a Specialty of Medicine, 526.
Dictated, But not Read, 537.
Die Metal, 429.
Differentiating Morphine, Codeine and Heroin, 634.
Disinfect for Dental Instruments 582.
Drilling Glass, 431.
Dr. Black Awarded the First Miller Memorial Medal, 431.
Duralumin, new Aluminum Alloy, 635.
Effect of Tobacco on Body and Mind, 548.
Electric Dental Engine, 636.
Electric Power from Solar, 211.
Extractions of Cuspids, 482.
Fees for Services rendered and not from Tradition, 101.
First Permanent Molars; their Treatment, 591.
Free School Dispensary Abroad. 107.
Gold Fillings in Porcelain Teeth 149.
Hard Gums, 592.
Hardening Plaster Casts, 535.
Herpes and Canker Sores, Dr. Pusey, 101.
High Pressure Anesthesia, 263.
History of Dental Dispensary, 105
Hot Air Therapy, 533.
How to Fix Fees, 537.
Inexpensive Sterilizer, 375.
Inlay Casting Flux, 374.
Insulating Cement, 374.
Is it Worth While, 104.
Keep at School, 587.
Keeping Impression Trays Clean 320.
Labeling Bottles, 636.
Law, 483.
Management of Patients, 482.
Masticating Test, 428.
Mastication with Full Dentures, 148.
Medical Knowledge, its Acquisition and Degree, 100.
Modern Dentistry and the Tuberculosis Problem, 451.
Need of Free Dental Service, 104.
New Method of Ether Anesthesia. 477.
New Principle in Difficult Bridge Work, 529.
Non-Enforcement of Anti-Spitting Ordinance, 588.
Oral Cavity of Maternity Nurses, 589.
Overcoming Brittleness of Gold Castings, 430.
Pain After Extraction, 583.

Pain from Tooth Extraction Fatal, 375.
Paraffin Root Filling, 52.
Paraform as an Obtundant, 259.
Peculiar Condition, 152.
Period in which Dental Operations may be Started, 262.
Plating Plant, 580.
Poison Ivy Remedy, 583.
Polishing the Proximal Surfaces, of Fillings, 262.
Porcelain Facings, 485.
Preventable Diseases, 637.
Prevention of Dark Joints, 148.
Prolonged Nitrous Oxid Anesthesia, 533.
Problem of Mastication, 537.
Prophylaxis for Infants, 638.
Purification of Wax, 429.
Purification of Water by Storage, 585.
Race Suicide Remedies, 637.
Removing Pulps, 430.
Removing Wax Inlay Impressions, 429.
Revocation of License to Practice, 625.
School Children's Teeth in New South Wales, 529.
School Clinics, 587.
Sciatica and Bridge Work, 538.
Sodoxylin, 633.
Soldering Fluid, 429.
Soldering Paste, 430.
Stearate of Zinc, 320.
Suction for Lower Dentures, 580.
Suggestions for Arranging Artificial Teeth, 149.
Surgical Treatment of Chronic Abscess, 479.
Sulfurous Acid in Dentistry, 534.
Suppurating Tooth Fatal, 320.
System of Cavity Preparation, 259.
Swedging Plates, 150.
The Chip Blower, 477.
The Dentist who is Past Fifty, 532.
The Dropper Ampoule, 213.
The Matrix, 260.
The Matrix in Gold Filling, 260.
The Scourge of the North, 52.
Toothache, 483.
Toothpowder, 483.
Treating Aphthea with Formol, 374.
Unhygienic Cigar Making, 588.
Unlawful Method of Collecting, 532.
Vaccine Treatment for Loose Teeth, 536.
Vulcanizer Alarm, 431.
Vulcanite Plate Repairing, 148.
Wax Dummies, 483.
Where the Poor Patients Come From, 102.
Zinc Dies, 149.

ANNOUNCEMENTS.

American Miller Memorial, 376.
Berlin Post Graduate School, 270.
Canadian Dental Association, 157, 269.
Doctor Black's Banquet, 54.
G. V. Black Dental Club Meeting, 640.
Illinois State Society, 157.
Indiana State Society, 267.
Institute of Dental Pedagogies, 639.
International Hygiene Exhibit, 377.
Iowa Dental Examiners, 216, 269, 594.
June Dental Meetings, 267 to 269
Kentucky State Dental Association, 157.
Marquette University Alumni Meeting, 640.
Michigan State Board of Examiners, 156, 216,. 539.
Michigan State Dental Society, 215, 376,
Michigan State Dental Society Meeting, 640.
National Association of Examiners 158, 267.
National Association of Dental Faculties, 266.
New Jersey State Examining Board, 157, 594.
New Jersey State Society, 215.
New York Seventh District Society, 540.
New Orleans Dental Association, 375.
New Orleans Odontological Society, 156.

Ohio Examining Board, 216, 269.
Ohio State Society, 539.
Oral Hygiene Convention in Cleveland, 159.
Southern Branch National Dental Association, 158.
The Miller Monument Fund, 214.
Tennessee State Association, 216.
Texas State Association, 53.
Utah Examining Board, 594.
Xi Psi Phi Fraternity, 486.

AUTOGRAPHIC.

Abbott, C. E., 576.
Allen, C. E., 477.
Bancroft, F. W., 384.
Barclay, A. C., 148.
Bernstein, E. J., 481.
Billow, G. A., 480.
Black, G. V., 261.
Brown, C. E., 149.
Brush, F. C., 141.
Buckley, F. S., 474.
Buckley, J. P., 142. 490.
Campbell, C. V., 320.
Carpenter, D. H., 485.
Cavanagh, W. B., 627.
Clapp, G. W., 29, 40, 127.
Cheney, W. E., 416.
Coats, J. A., 482.
Collins, W. H. Y., 521.
Conzett, J. V., 249.
Corrigan, M. P., 592.
Crawford, J. Y., 316.
Cross, H. A., 430.
Cryer, M. H., 347.
Duden, Alice, G. H., 502.
Engstead, J. E., 307.
Fletcher, M. H., 593.
Fenn, H. J., 581.
Fickes, W. L., 485.
Gotch, W. D., 167.
Gray, J. P., 148.
Gregg, Geo. T., 179.
Gritman, A. D., 149.
Harper, J. W., 187.
Harrower, H. R., 569.
Haskell, L. P., 136, 148, 153, 371, 471.
Hill, E. B., 483.
Hoff, N. S., 262.
Hubbard, Elbert, 324.
Hutchinson, R. G. Jr., 612.
Jackman, W. T., 263.
Johnson, C. N., 482.
Johnson, S. E., 374.
Jonesco, Dr., 153.
Jordan, L. A., 583.
Jungman, J. W., 482, 583.
Kern, R., 312.
Kirk, E. C., 639.
Knopp, S. A., 451.
Koch, C. R. E., 50.
Latham, Vida, A., 543.
Lewis, E. F., 219.
Lindstrom, C. R., 75.
Lodge, E. B., 262.
Lumbard, J. E., 567.
Masselink, B. H., 465.
McCarty, J. J., 560.
McCurdy, S. L., 546.
McCullough, P. B., 592.
Maerklein, B. J., 234.
Palmer, C. L., 439.
Parrott, A. H., 191.
Parsons, C. G., 304.
Pedley, J. R., 535.
Pogue, J. W., 17.
Polet, M., 204.
Potter, W. H., 57.
Poundstone, G. C., 459.
Power, J. E., 341.
Pritchett, H. S., 287.
Prothero, J. H., 112.
Pusey, Dr., 101.
Root, J. P., 517.
Rushton, Wm., 68.
Ruttloff, Carl, 455.
Schurman, J. G., 274.
Service, C. S., 625.
Smith, D. D., 597.
Smith, F. M., 413.
Starkweather, C. S., 430, 529.
Stevens, Alice M., 140, 532.
Stiff, F. W., 527.
Talbott, E. S., 329, 391.
Todd, Wm. H., 475.
Trueman, W. H., 5, 100, 537, 588.
Underwood, A. S., 538, 550.
Vallender, W. H., 529.
Warner, C. T., 409.
Watkins, S. C. G., 149.
Wallace, W. T., 151.
Weeks, T. E., 259, 312.

Wedelstaedt, E. K., 225.
West, J. B., 445.
Wheeler, H. L., 254.
White, P. C., 162.
White, P. G., 80.
Whitslar, W. H., 394, 583.
Williams, J. Leon, 260.
Wilson, G. H., 132.
Woods, J. A., 259.
Worthly, F. G., 150.
Young, D. H., 138.

### ANNOUNCEMENTS.

THE TEXAS STATE DENTAL ASSOCIATION. The annual meeting of the Texas State Dental Association will be held at Houston, Texas, May 10th, 1910. On May 11th, 12th and 13th the association will meet conjointly with the Southern Branch of the National Dental Association at the same place.

J. G. FIFE, *Secy*,
Dallas, Tex.

CELEBRATION IN HONOR OF DR. G. V. BLACK. The Chicago Odontographic Society have arranged for a great Meeting, Manufacturers Exhibit, Clinic and Banquet to be held on Friday and Saturday, January 28th and 29th, 1910, and a cordial invitation is hereby extended, to all ethical practitioners, to be present on these occasions.

Exhibitors Clinic, January 28th, 1910, all day (at the Chicago College of Dental Surgery, corner Wood and Harrison Sts.

Meeting, January 28th, 1910, at 8 P. M., in Handel Hall 40 E. Randolph St.

Essayist: C. N. Johnson, M.A., L.D.S., D.D.S., Subject: "The Selection of Filling Material and Methods of Inserting when Temporary work need not be considered." The discussion will be opened by prominent men from all sections of the country.

Clinics, Saturday, January 29th, 1910, all day at the Chicago College of Dental Surgery by operators of national reputation.

Banquet. As a fitting climax to a great meeting, we will all unite in a "Testimonial Banquet" to a great man, whom we all love to honor, Dr. G. V. Black. The banquet will be held in the Gold Room of the Congress Hotel on Saturday Evening, January 29th, 1910, at 7 o'clock. Information regarding meeting or banquet will be furnished by any officer of the society or by addressing the Clinic Committee, H. N. Orr, A. F. James, J. E. Schaefer, C. M. Cahill.

GEO. N. WEST, *Chairman*,
100 State St., Chicago.

